# Experimental and numerical analysis of flow through a natural rough fracture subject to normal loading

**DOI:** 10.1038/s41598-024-55751-w

**Published:** 2024-03-07

**Authors:** Paolo Trinchero, Liangchao Zou, Miquel de La Iglesia, Aitor Iraola, Patrick Bruines, Guido Deissmann

**Affiliations:** 1 AMPHOS 21 Consulting S.L., Carrer de Veneçuela, 103, 08019 Barcelona, Spain; 2 Department of Sustainable Development, Environmental Science and Engineering, KTH Royal Institute of Technology, 10044 Stockholm, Sweden; 3 Department of Teaching Mathematics and Experimental Sciences, Universitat Autònoma de Barcelona, 08019 Barcelona, Spain; 4 Swedish Nuclear Fuel and Waste Management Company, Box 3091, SE 169 03 Solna, Sweden; 5 Institute of Energy and Climate Research: Nuclear Waste Management (IEK-6) and JARA-CSD, Forschungszentrum Jülich GmbH, 52425 Jülich, Germany

**Keywords:** Hydrogeology, Geophysics, Fluid dynamics

## Abstract

Fractured crystalline rocks have been chosen or are under consideration by several countries as host rock formations for deep geological repositories for spent nuclear fuel. In such geological formations, flow and solute transport are mostly controlled by a network of connected natural fractures, each of them being characterised by internal heterogeneity, also denoted as roughness. Fractures are, in turn, subject to variable load caused by various factors, such as the presence of thick ice sheets formed during glaciation periods. Understanding how coupled hydro-mechanical (HM) processes affect flow and transport at the scale of a single natural fracture is crucial for a robust parameterisation of large-scale discrete fracture network models, which are not only used for nuclear waste disposal applications but are also of interest to problems related to geothermics, oil and gas production or groundwater remediation. In this work, we analyse and model an HM experiment carried out in a single natural fracture and use the results of both, the experimental and the modelling work, to get insights into fundamental questions such as the applicability of local cubic law or the effect of normal load on channeling. The initial fracture aperture was obtained from laser scanning of the two fracture surfaces and an equivalent initial aperture was then defined by moving the two fracture surfaces together and comparing the results obtained using a Navier–Stokes based computational fluid dynamics (CFD) model with the experimental flowrate obtained for unloaded conditions. The mechanical effect of the different loading stages was simulated using a high-resolution contact model. The different computed fracture apertures were then used to run groundwater flow simulations using a modified Reynolds equation. The results show that, without correction, local cubic law largely overestimates flowrates. Instead, we show that by explicitly acknowledging the difference between the mechanical aperture and the hydraulic aperture and setting the latter equal to 1/5 of the former, cubic law provides a very reasonable approximation of the experimental flowrates over the entire loading cycle. A positive correlation between fluid flow channeling and normal load is also found.

## Introduction

Various different natural subsurface formations, such as some igneous or metamorphic rocks, are characterised by both an intrinsically low permeability and a brittle behaviour. In such geological media the transport of fluids, energy and dissolved solutes primarily occurs through open or partly open fractures^1^, which typically display significant internal heterogeneity due to surface roughness. This heterogeneity is in turn dependent on in-situ stress conditions, which can change as a result of natural or anthropogenic processes. For example, in the context of deep geological disposal of spent nuclear fuel, long-term stress changes can be expected due to glacial advance/retreat^2^ and short-term changes can be caused by stress redistribution in the vicinity of the excavated access tunnels or by thermal stress leading to mechanical spalling in deposition holes^3^. Similar processes can be found in the context of other applications such as geothermal energy exploitation^4^, or storage of natural gas or hydrogen and geologic carbon sequestration^5^. Understanding and modelling the effect of these stress changes on fracture aperture is key to formulating reliable flow and transport models, which are needed for e.g. safety assessment studies for nuclear waste disposal or for optimising energy production in geothermal systems.

Flow through fractures has long been described using cubic law (CL)^6^, which is a straightforward expression that relates the volumetric flowrate with the fracture aperture. The CL was mathematically derived for a parallel plate fracture model^7^, i.e. for a simplified conceptual model assuming that the two fracture walls are perfectly smooth and parallel. The CL is also the basis for the Reynolds equation, also known as the local cubic law (LCL), which formulates the mass balance equation in terms of the local fracture aperture.

Several studies have pointed out that both the CL and the LCL are potentially unfit to model flow through natural rough fractures^8–10^. This inadequacy is partly related to violating the underlying assumptions (i.e. parallel and perfectly smooth fracture walls) and, for the LCL, partly caused by the sometimes ill-defined concept of local fracture aperture^9^. Several different and relatively complex correction methods have been proposed to alleviate such shortcomings based on numerical and analytical studies carried out on synthetically generated fracture apertures. In practical applications, such as the safety assessment study for the Finnish deep geological repository for spent nuclear fuel in Olkiluoto (TURVA 2020), often more pragmatic approaches have been used by, e.g., postulating the existence of different equivalent fracture apertures (e.g. mechanical, transport and hydraulic aperture), which are used in combination with the CL^11^. For instance, in the modelling work of TURVA 2020, mechanical and hydraulic aperture ($\delta _m$ and $\delta _h$ respectively) were assumed to be linearly related through a correction factor *f*, which is deemed to be in the range of 3–20 (i.e. ratio of hydraulic to mechanical aperture in the range of 0.05–0.3), based on estimations done using grout injection and flow logging test data^12^.

All the studies discussed in the previous paragraph do not explicitly account for mechanical processes such as variable normal loading conditions. Yet, a number of experimental and numerical studies have analysed the evolution of fracture openings as a function of externally applied normal forces. The earliest works on this topic proposed empirical models^13–16^ whose application was relatively straightforward but whose parameterisation remains largely uncertain. A more theoretical model, denoted as Greenwood–Williamson (GW) model, was later proposed based on the Hertzian contact theory for rock fractures^17,18^. Despite the underlying physical constraints, the GW model is still based on important simplifications since it neglects the interaction between nearby asperities and does not account for plastic deformation effects. A more general half-space approach was introduced by Tian and Bhushan^19^ based on Boussinesq’s equation, which relates contact stress to surface displacement of a semi-infinite body. The advantage of this so-called high-resolution contact model (HRCM), compared to the GW model, is that it does account for the interaction between adjacent asperities and can also consider local plastic deformation, which might be particularly important when modelling brittle materials such as crystalline rocks. HRCM was further validated by Zou et al.^20^ on a synthetic sample representative of crystalline rock.

In this work, we try to answer the question: “ how does water flow through a single natural rough fracture? ”. Several previous works have addressed or tried to address this question (see, e.g., the references above); however, this is done here using a novel methodological approach that involves the use of (1) experimental data from an HM experiment performed in a rock sample containing a natural fracture, (2) fracture scan data of the two fracture surfaces, (3) a Navier–Stokes CFD model, (4) an elastic-plastic contact model, and (5) a number of models based on the modified Reynolds equation. By incorporating a meticulously calibrated mechanical model in the modelling framework and by performing the validation over the full fracture loading cycle, the resulting considered modified Reynolds model, which assumes that the ratio between hydraulic and mechanical aperture is equal to 0.2, is deemed to provide a reliable description of flow through the considered deformable fracture. Although the generalisation of this modified Reynolds model is out of the scope of this work, the suggested ratio of the two apertures is consistent with the range provided by Hartley^12^. The effect of mechanical load on fluid flow channeling has also been investigated.

## Methods

The hydro-mechanical experiment assessed in this work was conducted as a part of the SKB Task Force on Modelling of Groundwater Flow and Transport of Solutes (GWFTS) and benefited from collaboration with POST-2. POST-2 was a large collaborative project, which was initiated in 2016 by two nuclear waste management organisations (SKB in Sweden and NWMO in Canada) and three universities and research centres (KTH, RISE and BEFO, all three located in Sweden).

### The sample and the hydro-mechanical experiment

**Figure 1:**
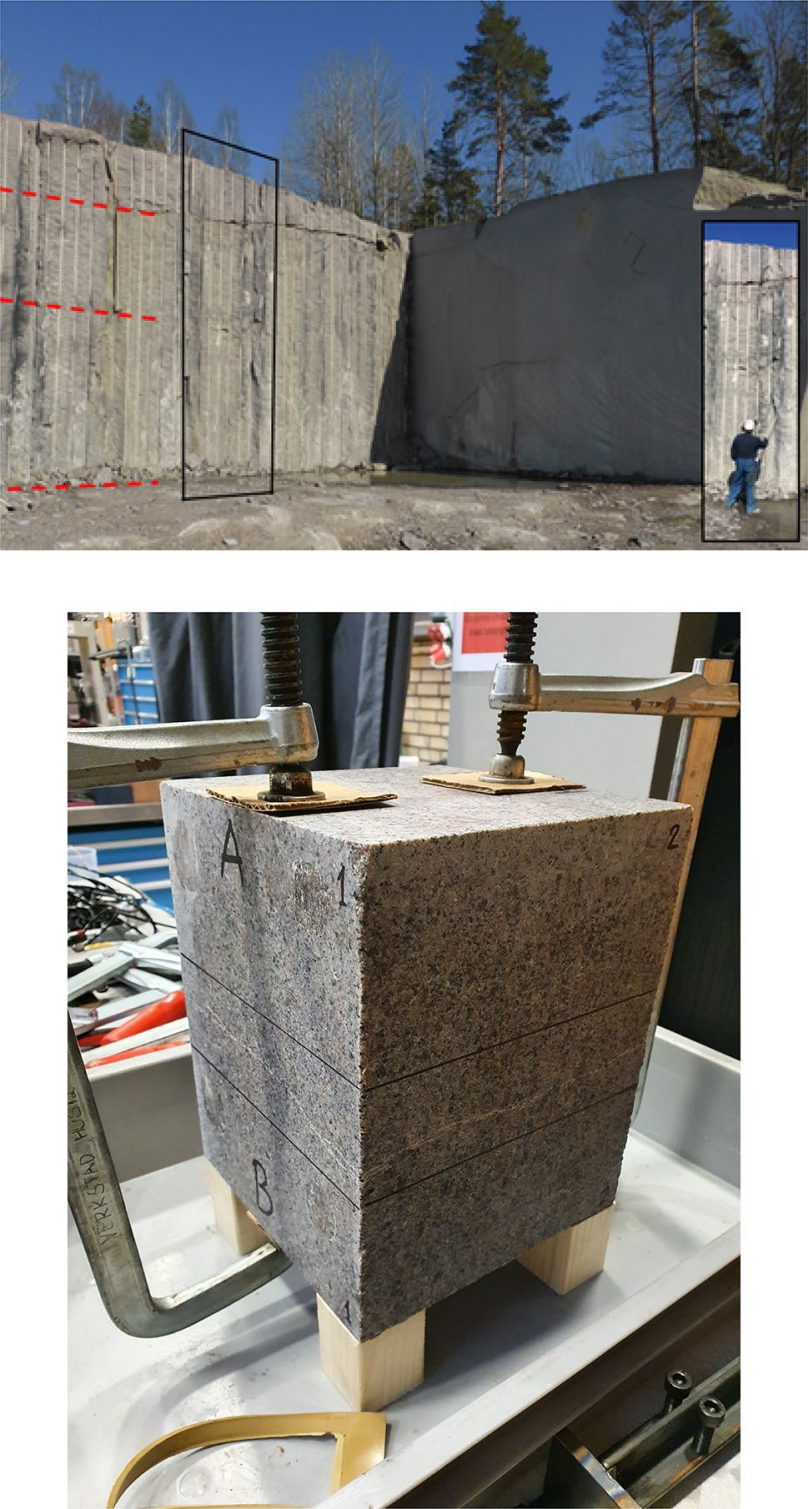
(Top) Location where the blocks were extracted in the Flivik quarry in Oskarshamn municipality, Sweden. The red lines show horizontal exfoliation joints. The small picture insert shows the size of the wall. The vertical wall having drill holes and blasting surface is the hardway plane and the smooth wire cut surface is in approximately along the grain plane. (Bottom) Detailed view of the specimen used in the HM experiments. The two slots were used to place the lateral rubber band. Figures courtesy of RISE^33^.

The specimen used in the experiment is a rectangular rock block with dimensions 200 mm by 200 mm by 250 mm, as shown in Fig. 1. The rock sample was cut from a 19.6 tonne block of medium to fine-grained granite. The block was quarried from Flivik quarry in Oskarshamn municipality, in Sweden (Fig.  1) that belongs to the 1.8–1.7 Ga Transscandinavian Igneous Belt (TIB). The natural fracture was originally oriented as an almost vertical fracture in the quarry but was cut to be as parallel as possible to the upper and lower surface of the specimen, i.e. to achieve a square fracture of 200 mm by 200 mm (Fig.  1).

**Figure 2:**
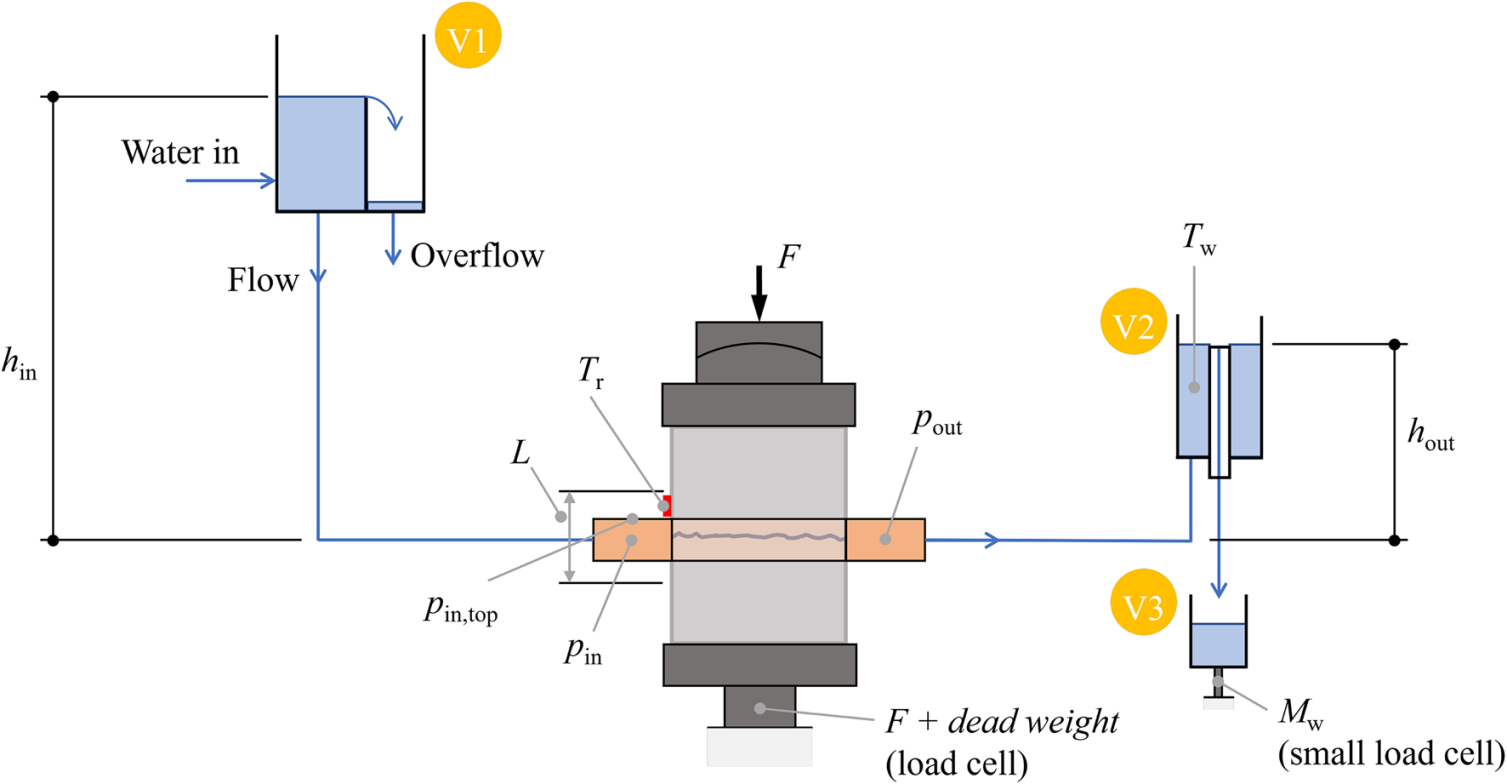
Sketch showing the main components of the hydro-mechanical experiment. V1, V2 and V3 are, respectively, the inlet vessel (with constant head level $h_{in}$), the outlet vessel (with constant head level $h_{out}$) and the collection vessel. $M_w$ is the water mass recorded in the load cell connected to the collection vessel, $T_w$ and $T_r$ are, respectively, the temperature in the water measured in vessel V2 and the temperature measured in the rock close to the inflow, *F* is the normal compression force, $p_{in}$ and $p_{in,top}$ are the water pressures recorded in the inlet chamber, $p_{out}$ is the water pressure recorded in the outlet chamber and *L* is the distance over which the deformation of the rock sample is measured.

A sketch of the hydro-mechanical experiment is shown in Fig. 2. The setup was configured to conduct a constant head boundary hydraulic test. Hence, constant head was applied on the inflow and outflow sides of the fracture whilst the remaining two sides were sealed, following the setup from upstream to downstream. Different constant head boundaries were achieved by hoisting a vessel (V1 in Fig.  2) with an overflow mechanism to different altitudes above the fracture. The vessel V1 was hydraulically connected, using a hose with diameter of 8.5 mm, to the inlet chamber at the upper boundary. The inlet chamber was 40 mm high, 200 mm wide and 120 mm deep to ensure the head was constant along the fracture. Ideally, with a frictionless hose, the pressure is equal to $p_{in} = \rho g H_{in}$ (Pa). However, in reality, there is a loss, and hence, the pressure is measured in the inlet chamber, instead of using the head from the inlet overflow. The sealing along the no-flow boundaries was simply done by pressing a soft rubber band against the fracture outlet. On the downstream boundary, an outlet chamber identical to the inlet chamber with a pressure gauge was attached to ensure the head was equal along the fracture. The outlet chamber was hydraulically connected to the overflow vessel, V2 in Fig.  2, using another hose with a diameter of 8.5 mm. The overflow vessel was introduced to achieve a head boundary as constant as possible. Effects of surface tension in the water were minimized using a PTFE-plastic pipe as the overflow. The overflowing water was collected in a vessel, V3 in Fig. 2, where the mass of the water was continuously monitored. This mass rate is used to infer the flowrate through the fracture. To investigate how the flowrate varies by normal stress, the specimen was inserted in a hydro-mechanical device. For the unopened fracture, two loading-unloading cycles, in steps as follows; 0.1, 1, 2, 4, 8, 4, 2, 1, and 0.1 MPa, were applied. Only the first loading cycle is considered in this study. The compression of the fracture was recorded using four linear variable differential transformers (LVDT) attached to frames mounted to the specimen. At each stress state, multiple heads were applied, and the mass flowrate was recorded for each setup. Hence, at each stress level and head applied, the following measures were recorded:

- Normal load applied by the hydraulic device (kN);
- Displacement from the four LVDT sensors (mm);
- Pressures at the fracture inlet and outlet ($p_{in}$ and $p_{out}$) (Pa)
- Temperature of water and rock at the inlet (°C);
- Temperature of water and rock at the overflow vessel (°C);
- Force at the load cell of the outflow vessel V3 (N)

Table S1 in Supplementary Material provides all the values measured during the first loading-unloading cycle.

**Figure 3:**
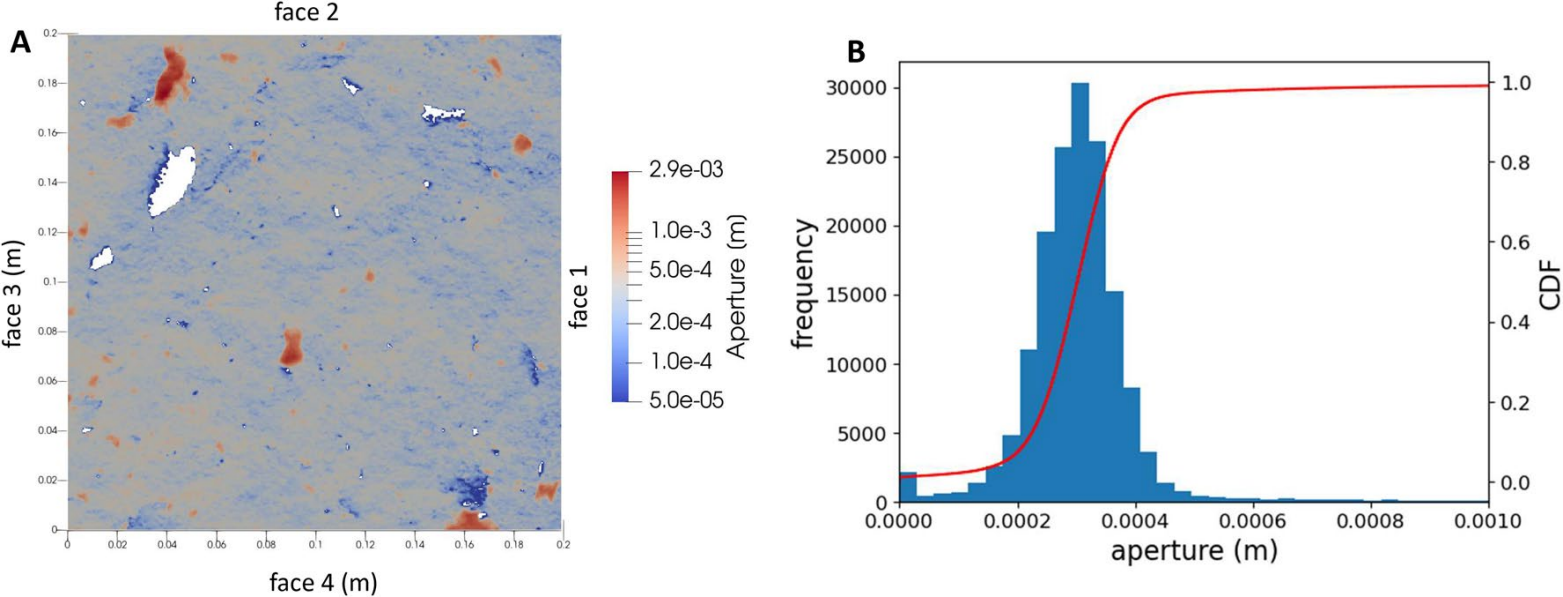
(**A**) Initial fracture aperture estimated from the upper and lower fracture scans. White regions are contact points. (**B**) Histogram and cumulative distribution function (CDF) of the initial fracture aperture.

After the first two loading-unloading cycles, the fracture was opened for fracture surface characterisation. To open the fracture, a force had to be applied on a specially designed device. It was estimated that a moment of approximately 100 Nm had to be applied to break the remaining rock bridges between the fracture surfaces. Only a few slivers detached from the fracture wall during the operation. The two fracture surfaces (noted as upper and lower surfaces from now on) were subsequently scanned using a handheld laser scanner (Leica LAS) in combination with the Leica Absolute Tracker AT960, with a resolution of 0.1–0.2 mm. The used system has a nominal precision of 0.035 mm. Besides fracture surfaces, the sides of the sample were also scanned so that both fracture surfaces could be referenced back to the original reference system. The initial fracture aperture, estimated as the difference between the upper and the lower fracture surface heights, along with the related statistical parameters, is shown in Fig.  3. Note that during the first loading-unloading cycle, the flow was from face 2 to 4. It is worth noting that the reddish areas in the figure mostly result from flakes or fragments coming loose when the fracture was broken apart. The impact of these areas on flow is deemed minor since they do not form continuous paths but rather isolated spots.

### The models based on the Navier–Stokes equations

The numerical simulations based on the Navier–Stokes (NS) equations were carried out using the open-source computer code OpenFOAM. The fracture void space was first reconstructed using the fracture surface data and later meshed using one of OpenFOAM’s internal mesh generators called snappyHexMesh. Meshing was done with great care in order to ensure consistency between the original aperture fields and the corresponding representation in the computational mesh. This check was done by comparing the corresponding histograms of fracture aperture (Figure S1 in Suppementary Material).

The governing equations solved in the model are the NS equations (conservation of momentum and mass) for incompressible flow:

1 $$\begin{aligned} \rho \left( \frac{\partial \textbf{u}}{\partial t}+ \left( \textbf{u} \cdot \nabla \right) \textbf{u} \right) =-\nabla p+\mu \nabla \textbf{u} \end{aligned}$$

2 $$\begin{aligned} \nabla \cdot \textbf{u}=0 \end{aligned}$$

where $\textbf{u}$ (m s$^{-1}$) is the water velocity, $\rho$ (kg m$^{-3}$) is the water density, $\mu$ (Pa s) is the water viscosity and p (Pa) is the water pressure.

The global Reynolds number of a given simulation can be computed as:

3 $$\begin{aligned} Re=\frac{\rho Q}{\mu W} \end{aligned}$$

where *Q* (m$^3$ s$^{-1}$) is the flowrate and *W* (m) is the fracture width, equal to 0.2 m.

### The elastic-plastic contact model

In this sub-section, we briefly summarise the equations of the elastic-plastic contact model used in this work to simulate the mechanical processes. For more details of implementation and validation, the reader is referred to Tian and Bushan^19^ and Zou et al.^20^.

For an elastic contact problem of two rough elastic surfaces, the total complementary potential energy, $V^*$, is given by^19^,

4 $$\begin{aligned} V^*=\frac{1}{2} \iint _{\mathbb {A}} \sigma _{\textrm{n}} (x,y) {\overline{u}}_z (x,y)dx dy - \iint _{\mathbb {A}} \sigma _{\textrm{n}}(x,y) {\overline{u}}_z^* dx dy \end{aligned}$$

where ${\mathbb {A}}$ (m$^2$) is the domain of contact surface, $\sigma _{\textrm{n}}(x,y)$ (Pa) is the normal stress, ${\overline{u}}_z (x,y)$ (m) is the composite surface normal displacement in the contact area $\mathbb {A}$ (m$^2$) to be calculated, and ${\overline{u}}_z^*$ (m) is the total prescribed displacement of the two contacting surfaces in the assumed contact area based on geometrical interference. The ${\overline{u}}_z (x,y)$ is related with $\sigma _{\textrm{n}}(x,y)$ by using Boussinesq’s solution for surface displacement subject to normal distributed load on the surface of a semi-infinite elastic body, expressed as a Green’s function.

5 $$\begin{aligned} {\overline{u}}_z (x,y) = \iint _{\mathbb {A}} A_{zz} (x,y,\xi ,\eta ) \sigma _{\textrm{n}} (\xi ,\eta ) d\xi d\eta \end{aligned}$$

where $A_{zz}(x,y,\xi ,\eta )$ is the influence function for normal deformation at the location (*x*, *y*), due to a unit stress at the position $(\xi ,\eta )$. It is calculated by

6 $$\begin{aligned} A_{zz} (x,y,\xi ,\eta ) = \frac{2(1-\nu ^2)}{\pi E \sqrt{(x-\xi )^2+(y-\eta )^2}} \end{aligned}$$

where $\nu$ is the Poisson’s ratio and *E* (Pa) is the Young’s modulus.

In this contact model, the normal stress causing plastic deformation is considered by assuming that the rock asperity deforms as an elastic-perfectly plastic material and the plastic deformation is confined within a small local area. In this case, the dissipation of energy caused by plastic deformation is added to the total complementary potential energy, written as^19^

7 $$\begin{aligned} V^*=\frac{1}{2} \iint _{\mathbb {A}} \sigma _{\textrm{n}}(x,y) {\overline{u}}_z (x,y)dxdy - \iint _{\mathbb {A}} \sigma _{\textrm{n}} [{\overline{u}}_z^* -\frac{1}{2} \Delta u_z^{\textrm{p}}(x,y)]dxdy \end{aligned}$$

where $\Delta u_z^{\textrm{p}}(x,y)$ (m) is the composite incremental surface displacement in the plastic deformation zone where the contact stress reaches the hardness of the rock, *H* (Pa), i.e., $\sigma _{\textrm{n}}(x,y)>H$. The hardness, *H*, is a parameter describing the resistance against non-elastic deformation of the rock. The $\Delta u_z^{\textrm{p}}(x,y)$ is given by^19^

8 $$\begin{aligned} \Delta u_z^{\textrm{p}}(x,y)=\frac{1}{2} {\overline{u}}_z^p (x,y) \end{aligned}$$

where ${\overline{u}}_z^p (x,y)$ (m) is the incremental displacement of two contact surfaces where contact stress reaches the rock hardness. The discretization and numerical strategy for these equations, as well as experimental validation, can be found in Zou et al.^20^.

### The models based on the modified local cubic law

In this set of models, groundwater flow is described using the 2-D steady-state flow equation:

9 $$\begin{aligned} \nabla \cdot \left( T \nabla h \right) =0 \end{aligned}$$

where *T* (m$^2$ s$^{-1}$) is the transmissivity and *h* (m) is the hydraulic head.

The CL establishes a simple relationship between fracture aperture ($\delta$ (m)) and transmissivity^6^:

10 $$\begin{aligned} T=\frac{\delta ^3 \rho g}{12 \mu } \end{aligned}$$

We have already discussed in the introduction that the CL has been mathematically derived for a parallel-plate model and that its application for the modelling of flow through natural rough fractures leads to a significant overestimation of flowrates. Here, this is assessed by using a modified transmissivity vs. aperture relationship:

11 $$\begin{aligned} T=\frac{\left( A \cdot \delta _m\right) ^B \rho g}{12 \mu } \end{aligned}$$

In this modified relationship, the subscript *m* has been used to denote the mechanical aperture, which is defined as the distance between the two fracture walls, and *A* is a constant with units (m$^{3-B}$). When $B=3$, *A* should be seen as a scaling factor that relates the mechanical aperture to the hydraulic aperture ($\delta _h$). Different equivalent fracture apertures have been proposed in the context of several previous studies^21,22^.

By combining Eqs. (9) and (11), the conservation of mass can be written as:

12 $$\begin{aligned} \nabla \cdot \left( \delta _m^B \nabla h \right) =0 \end{aligned}$$

which can be seen as a modified Reynolds equation.

The models based on the modified LCL were run using the finite difference numerical code MODFLOW2000^23^.

### Modelling workflow

The modelling workflow follows a stepwise methodology:

- The first step consists of inferring the spatial distribution of the local fracture aperture. In the absence of direct measurements of this parameter, which could be made using micro-CT scanning, for example, the local aperture is estimated here from the fracture scan data, i.e. from measurements of the topography of the upper and lower fracture surfaces. These measurements do not provide a direct estimate of the local aperture, but when the data are referenced back to the same reference system, the difference between the upper and lower surfaces provides an estimate of the local aperture. This was done by first interpolating the fracture surface topography data of both the upper and lower fracture surfaces in a regular grid of size 0.5 $\times$ 10$^{-4}$ m by 0.5 $\times$ 10$^{-4}$ m and then obtaining the aperture as the difference between the two. The interpolation was performed using the nearest-neighbour method. All the subsequent modelling steps were performed based on this regular grid.
- The reconstructed fracture aperture (previous bullet point) was used to set-up a NS-based CFD model. As discussed in “Results” section, this model significantly overestimates the observed flowrates, requiring the definition of an equivalent initial aperture (next step).
- Trial and error calibration was carried out by progressively shifting one of the two fracture surfaces and solving the NS equation on the reconstructed 3D fracture volume. This was done in successive steps until reasonably matching the initial flowrate (i.e. unloaded conditions in the first loading-unloading cycle). The results of the numerical analysis provide an equivalent fracture aperture that reproduces the flowrate observed for unloaded conditions while still preserving the underlying roughness characteristics. This modelling step is discussed more in detail in “Results” section.
- The contact model was run and used to compute apertures at the different loading states, from 0 to 8 MPa. Only the loading stage was considered.
- Equation (11) was used to map the initial and final fracture aperture into related transmissivity fields and the modified LCL is used to estimate flowrates for the initial and final aperture and a range of *A* and *B* parameters.
- By analysing the mean squared error (MSE), computed based on the difference between the simulated and the experimental flowrates at the initial (unloaded state) and final stage (load of 8 MPa), two different pairs of (*A*,*B*), were selected and the corresponding fluid flow simulations were run for the full loading cycle using the modified LCL and the aperture fields computed by the high-resolution contact model. The resulting flowrate vs. load curve was compared with the corresponding experimental results.

## Results

### Definition of an initial equivalent fracture aperture

The first NS-based simulation was carried out using a fracture volume reconstructed from the initial fracture aperture (Fig. 3A). The resulting computational mesh consists of 52 million elements with a higher refinement close to contact points. An upwind second-order difference scheme is used for both velocity and pressure^24^. The PIMPLE (pressure-implicit with splitting of operators, semi-implicit method for pressure-linked equations) solver is used for the calculations. PIMPLE uses a semi-implicit approach to handle the pressure–velocity coupling and employs a pressure-correction approach to ensure that the pressure and velocity fields are consistent with each other. In each time step, the solver alternates between solving for a velocity predictor field and correcting the pressure field. These steps are repeated until the solution converges to a certain criterion. The linear solver employed was the geometric-algebraic multi-grid (GAMG).

Constant pressure was prescribed at face 2 ($p_{in}$) and face 4 ($p_{out}$) boundaries, with $\Delta p=p_{in}-p_{out}=3.9 \times 10^4$ Pa and a parabolic profile of velocity was assumed at the boundaries. The other two boundary faces (face 1 and 3) and the fracture walls were specified as no-slip boundaries. Water density and viscosity were set equal to 1000 kg m$^{-3}$ and $1.0 \times 10^{-3}$ Pa s, respectively. This and all the other NS-based calculations were run in the supercomputer JURECA of the Jülich Supercomputing Centre^25^ using 256 processor cores. The resulting computed flowrate was $3.8 \times 10^{-6}$ m$^{3}$ s$^{-1}$, which gives $Re=19$. Given this high Reynolds number, an additional simulation was run by including a turbulent model based on Reynolds-averaged Navier–Stokes equations and a k-omega shear stress transport model implemented in the pimpleFoam solver from OpenFOAM^26^. The resulting computed flowrate was slightly lower ($6.0 \times 10^{-7}$ m$^{3}$ s$^{-1}$). Overall, the experimental flowrate observed for unloaded conditions (Table S1 in Supplementary Material) was overestimated by a factor 11 by the turbulent model and a factor 70 by the laminar model.

There are several possible reasons for the overestimation of the flowrate mentioned above. One of these is that the fracture had to be opened to perform the scanning and, despite the great care taken in this operation, some material loss was reported, which does indeed have some impact on the reconstruction of initial aperture. Furthermore, after the manual laser scanning, carried out with a device with nominal precision of 0.035 mm, the two fracture surfaces had to be referenced to the original reference system and this step required some manual fitting, which is indeed prone to error. Finally, the high pressure at the inlet may have caused some unexpected effects such as slight lateral offset or rotation, particularly during the experiment carried out under unloaded conditions. The exact quantification of these additive effects is difficult if not impossible. We have therefore decided to take a different approach here, which is to define an equivalent, but realistic, fracture aperture capable of explaining the observed flowrate under unloaded conditions. This will be the starting point for the subsequent hydro-mechanical analysis. The definition of this initial equivalent aperture was done by manual trial and error tuning by (1) shifting one of the two fracture surfaces perpendicularly to the other, (2) reconstructing the fracture volume and meshing it and (3) solving the NS equation and computing the total flowrate. The advantage of this approach is that the resulting equivalent fracture aperture preserves the roughness features of the scanned fracture surfaces reasonably well.

**Figure 4:**
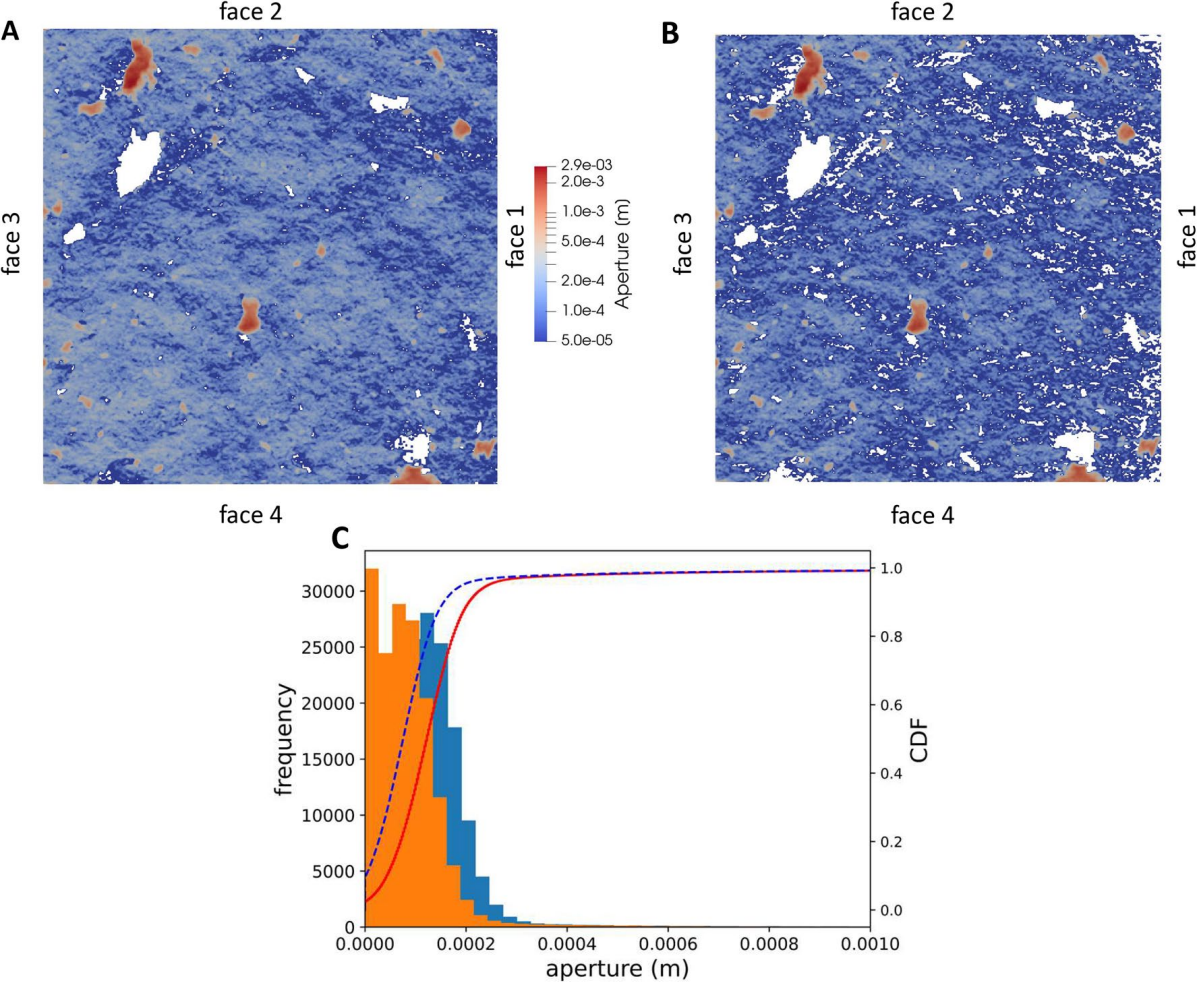
(**A**) Initial calibrated fracture aperture. (**B**) Fracture aperture at 8 MPa loading stage (i.e. final aperture). In both A and B, dark blue regions have an aperture value of $\le$ 5 $\times$ 10$^{-5}$ m. (**C**) Histogram and cumulative distribution function (CDF) for the initial calibrated fracture (blue histogram and red continuous line) and the final aperture (orange histogram and dashed blue line).

**Figure 5:**
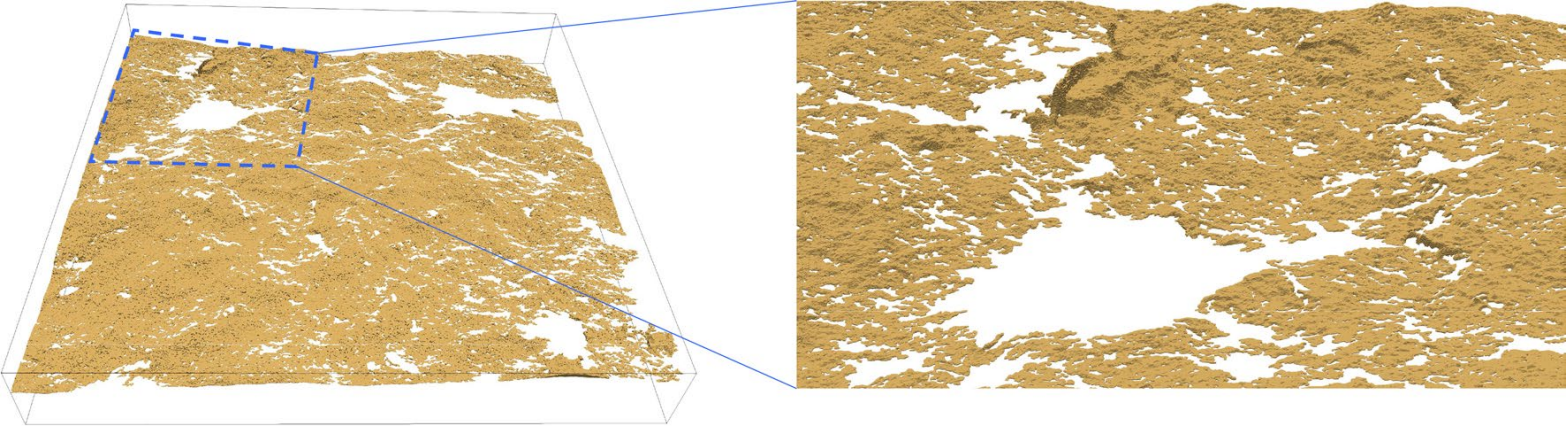
Details of the computational grid used for the Navier–Stokes simulations with 32 million elements.

The final equivalent initial aperture was obtained with a shift of 0.18 mm (Fig.  4A). A careful check of the snappyHexMesh discretisation was carried out to ensure that the fracture aperture was correctly captured by the CFD model (Fig. S1 in Supplementary Material). The resulting flowrate, estimated using an NS simulation (Fig.  5), was $5.3 \times 10^{-8}$ m$^{3}$ s$^{-1}$, which is inline with experimental results. The resulting Reynolds number is low ($Re=0.3$), which confirms that, with the considered calibrated fracture aperture, the flow regime is indeed laminar. It is worthwhile noting that the calibrated fracture aperture (Fig.  4A) and the computational grid (Fig. 5) are visually different. This is because in the computational grid unconnected cells and regions with extremely narrow aperture ($\le$ 5 $\times$ 10$^{-5}$ m) are removed. The comparison of the CDF distributions of the initial fracture aperture and the discretised initial fracture aperture (Fig. S1 in Supplementary Material) shows that the bulk of the distribution is well captured by the computational grid of the CFD model, with small discrepancies observed only for the smaller percentile (i.e. the smallest aperture regions).

### Mechanical modelling

The high-resolution contact model described in “Methods” section was used to calibrate and simulate the loading stage. Equal Young’s moduli ($E_1=E_2=E$) and Poisson ratios ($\nu _1=\nu _2=\nu$) were used for the upper and lower fracture surfaces, which were set equal to 73 GPa and 0.29, respectively, measured in POST project^27^.

The loading stage considered a maximum load of 8 MPa that was reached using 20 numerical steps. The hardness coefficient was manually calibrated based on the measured normal displacement data and set equal to $H=91.33$ MPa. Note that the hardness coefficient was the only parameter calibrated using the normal loading test data.

**Figure 6:**
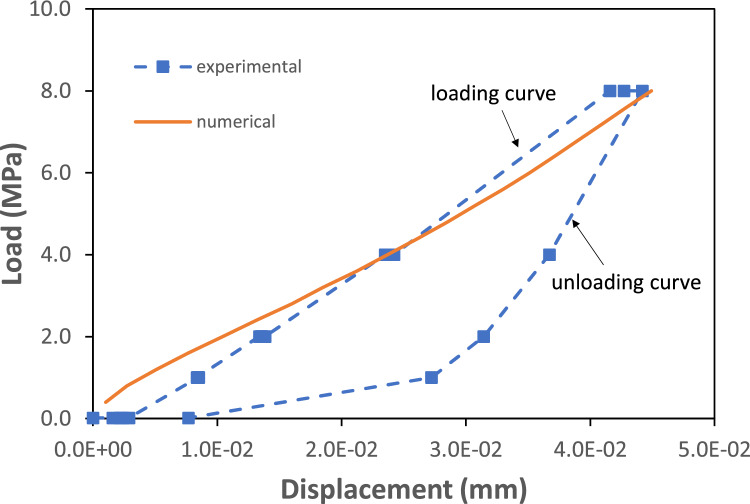
Displacement vs. load as computed by the high resolution contact model (continuous line) and average experimental values registered by the four LVDT sensors (dashed line). The average experimental series was centered by subtracting the first value from all data points.

Figure 6 shows the comparison between the average computed displacements vs. the average values registered by the four LVDT sensors. The model reproduces well the loading part of the hydro-mechanical experiment. The resulting final fracture aperture (i.e. fracture aperture at the end of the loading stage) is shown in Fig.  4B. By visually comparing Fig. 4A,B, it is evident that loading has a significant impact on the fracture void volume: besides an increase of contact points, there is also a significant increase of areas of aperture smaller than 5 $\times$ 10$^{-5}$ m, that are zones which have a second-order effect on flowrates. It is also interesting to compare statistics of fracture aperture at the beginning and the final stage of the loading part of the experiments. These are shown in the form of histograms and cumulative density functions in Fig. 4C and summarised in terms of different percentiles in Table 1. At the end of the loading stage (i.e. 8 MPa normal load) the 25th percentile (i.e.first quantile) is reduced by 55%, the median by 46%, the third quantile by 31%, and the 99th percentile by 4.7%. The relative standard deviation (RSD) values are presented in Table 1 to quantify the changes in the roughness characteristics of the fracture. The RSD is the ratio between the standard deviation and mean value of the fracture aperture. The RSD of the initial aperture is much larger than the aperture from scan data, and the RSD increases with the normal loading. Nevertheless, the roughness characteristics of the fracture surfaces are relatively well preserved in the equivalent initial fracture aperture and the normal loading process. The root mean square of the first derivatives of asperity heights ($Z_2$) for the upper and lower surfaces are 0.28 and 0.29, respectively, which nearly stay constant in the equivalent initial aperture and during the normal loading.

**Table 1:** Different percentiles of the fracture aperture distribution at the beginning (initial aperture, third column) and end of the loading stage (final aperture, fourth column).

	Aperture from scan data	Initial aperture	Final aperture
$P_{25}$ (m)	$2.6\times 10^{-4}$	$8.0\times 10^{-5}$	$3.6\times 10^{-5}$
$P_{50}$ (m)	$3.0\times 10^{-4}$	$1.2\times 10^{-4}$	$7.5\times 10^{-5}$
$P_{75}$ (m)	$3.4\times 10^{-4}$	$1.6\times 10^{-4}$	$1.1\times 10^{-4}$
$P_{99}$ (m)	$1.0\times 10^{-3}$	$8.5\times 10^{-4}$	$8.1\times 10^{-4}$
Contact points (%)	1.1	2.5	9.8
RSD (-)	0.5	1.0	1.4

The percentage of contact areas is also provided. The second column shows the different percentiles of the aperture as estimated from the fracture scan data (i.e. before calibration).

### Modelling of flow rates based on the modified local cubic law

The initial and final fracture aperture is used here to compute estimates of water flowrates (i.e. water flowing in and out of the fracture). We recall that ‘initial fracture aperture’ refers to the aperture for unloaded conditions, whereas ‘final aperture’ is the aperture obtained at the end of the first loading cycle when a nominal load of 8 MPa is applied to the sample. The former is computed from the scanned fracture surface data, which were calibrated using the CFD model, whereas the latter is obtained from the high-resolution contact model.

The modified Reynolds equation (Eq. 12) is solved by prescribing constant hydraulic head to face 2 (inlet) and face 4 (outlet) boundaries with a gradient of 20.3 (–). Parameter *A* is varied over a range [0.1, 1] with a discretisation step of 0.1; for parameter *B* a range [2, 4], with a discretisation step of 0.2, is considered. It has already been discussed in “Methods” section that parameter *A* does not influence the modified Reynolds equation, which only depends on *B*. Thus, strictly speaking, the numerical simulations related to the considered range of variability of *A* are redundant since for two given simulations, *i* and *j*, based on the same exponent *B* and two different *A* values ($A_i$ and $A_j$, respectively), the ratio of the two corresponding flowrates is given by $Q_i/Q_j=\left( A_i/A_j \right) ^B$.

**Figure 7:**
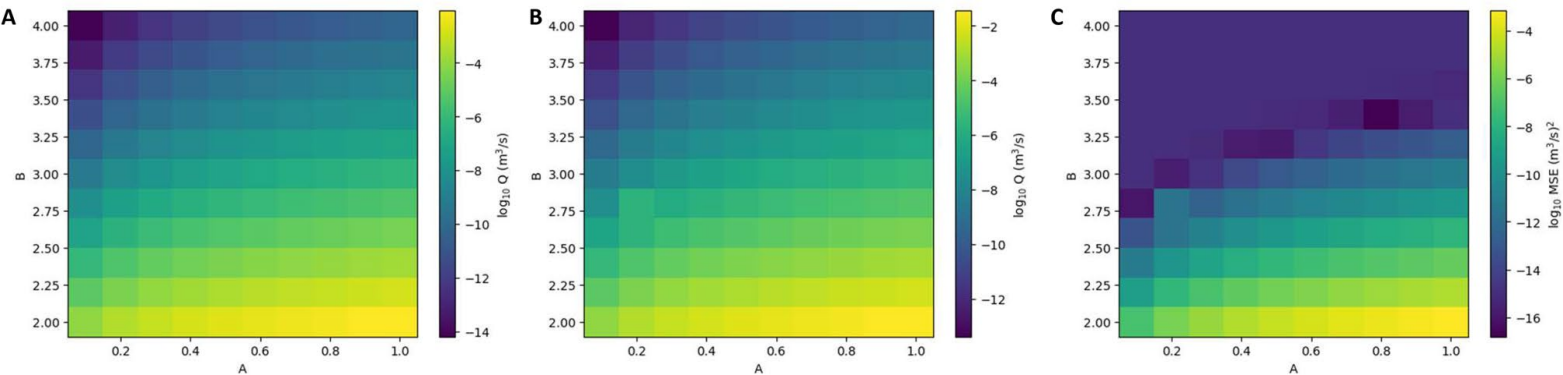
Logarithm with base 10 of the flowrate, *Q*, computed using the modified cubic law based on (**A**) the initial aperture and (**B**) the final aperture at 8 MPa load. (**C**) Mean squared error, MSE, based on the initial and final flowrate estimation. The initial and final experimental flowrates are, respectively, $5.8\times 10^{-8}$ m$^3$ s$^{-1}$ and $3.8\times 10^{-9}$ m$^3$ s$^{-1}$.

Figure 7A,B show the logarithm with base 10 of the flowrate, *Q*, computed using the initial and the final fracture aperture (we recall that the initial experimental flowrate is $Q \approx 5 \times 10^{-8}$ m$^3$ s$^{-1}$ whereas the final flowrate is $Q \approx 3.8 \times 10^{-9}$ m$^3$ s$^{-1}$). Figure 7C shows the mean squared error, computed based on the difference between the simulated and the experimental flowrates at the initial (unloaded state) and final stage (load of 8 MPa). From this figure, it is evident that there is a range of pairs [*A*, *B*] that gives average errors close to or smaller than $1.0 \times 10^{-8}$ m$^3$ s$^{-1}$. Based on these results, two pairs [*A*, *B*] have been selected for further analysis: [0.8, 3.4] and [0.2, 3.0]. The former has been chosen since it is the global minimum, whereas the latter has been selected because it is the pair that gives the minimum mean squared error (MSE) among all the considered cases with exponent 3.0. The case based on pair [1.0, 3] is also further considered since this corresponds to a simulation where the hydraulic aperture is set equal to the mechanical aperture, and the LCL is considered. For simplicity, this additional case will be denoted from now on as *cubic law* case.

**Figure 8:**
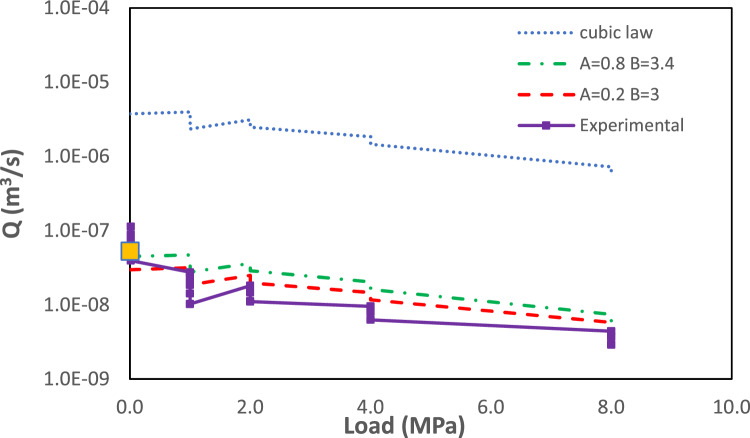
Experimental flowrates (continuous line) vs. normal load computed using two different pairs of values [A, B] for the modified local cubic law. The results of a simulation where the hydraulic aperture has been set equal to mechanical aperture and cubic law is considered, are also shown with a dotted line (*cubic law* in the legend). The orange dot at 0 MPa is the flowrate computed using the Navier–Stokes equations.

The three selected realisations were used to reproduce the evolution of flowrates during the loading cycle. This included a first loading step at 1 MPa (with the hydraulic gradient sequentially decreasing from 24.9 to 22.3, 19.7, 17.2 and finally 14.7), a second step at 2 MPa (with the hydraulic gradient sequentially decreased from 24.8 to 22.3 and 19.8), a third step at 4 MPa and a last step of 8 MPa (in these two last steps the same sequence of head gradients was used as in the 2 MPa step). Fracture apertures at the different considered loading steps were provided by the mechanical model (see previous sub-section). The computed vs. experimental flowrates are shown in Fig.  8. From the results, it is evident that, when directly used with mechanical aperture, CL overestimates flowrates by two orders of magnitude. When the aperture is reduced by 80%, and the CL is still considered (i.e pair [0.2, 3.0]), a quite good agreement with experimental results is obtained for the entire loading cycle. The agreement obtained with pair [0.8, 3.4] is also very good.

The simulation case considered above and based on the pair [0.2, 3.0] is based on the LCL and assumes that the ratio of hydraulic to mechanical aperture is equal to 0.2. This value is within the previously discussed range estimated from in-situ experiments at the Olkiluoto site.

**Figure 9:**
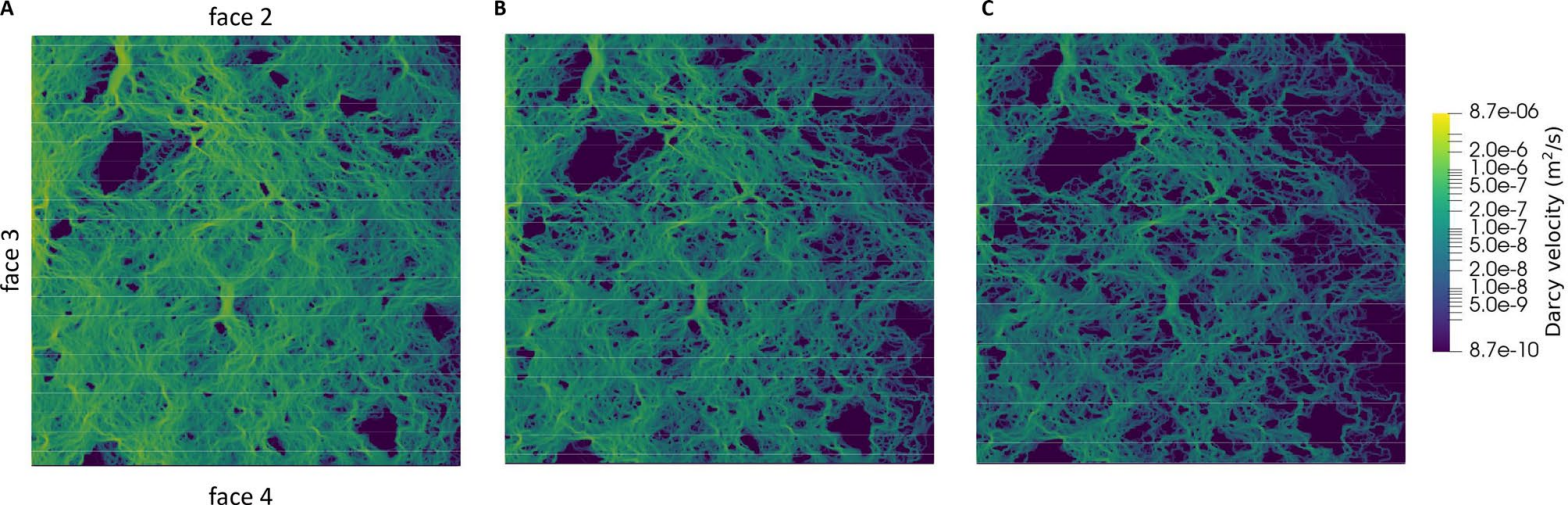
Magnitude of Darcy velocity computed using the modified local cubic law model with [0.2, 3] for (**A**) the initial fracture aperture, (**B**) fracture aperture at 4 MPa loading and (**C**) fracture aperture at the end of the loading stage (8 MPa).

**Figure 10:**
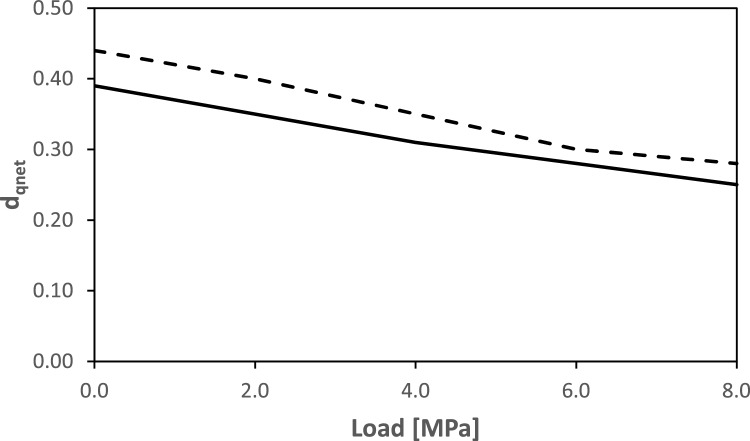
$d_{qnet}$ computed for the entire loading stage using the modified cubic law with pair [0.8, 3.4] (continuous line) and pair [0.2, 3.0] (dashed line).

It is also interesting to assess the influence of both the heterogeneous aperture field and the applied mechanical processes on flow channeling. The latter is here defined as the phenomenon by which liquid flow occurs preferentially in a limited area of the fracture^28^. Notice that channeling depends on the distribution of flux, not velocity. Thus, a first visual analysis can be done by looking at the distribution of the magnitude of Darcy’s velocity at different loading stages. This is shown in Fig.  9 for the LCL model based on pair [0.8, 3.4] (similar results are obtained for pair [0.3, 3] and are not shown here for the sake of brevity). The plots show that a certain degree of channeling exists for unloaded conditions as a result of the internal heterogeneity of the fracture. This channeling is, however, significantly increased when mechanical load is applied. In other words, even if the fracture at the end of the loading cycle transmits less water (Fig.  8), this is significantly more channeled. This can be quantitatively confirmed by using the following channeling indicator:

13 $$\begin{aligned} d_{qnet}=\frac{1}{\sum A_i}\frac{\left( \sum A_i \cdot |\mathbf {q_i} |\right) ^2}{\left( \sum A_i \cdot |\mathbf {q_i} |^2 \right) } \end{aligned}$$

where $A_i$ (m$^2$) is the area of *i*-th grid element and $\mathbf {q_i}$ (m$^2 s^{-1}$) is the corresponding Darcy velocity. Equation (13) is a slightly modified version of the original channeling indicator that was proposed by Maillot et al.^29^ for a network of fractures. Here, $d_{qnet}$ is to be intended as the fraction of the fracture that carries more water^30^. $d_{qnet}$ as a function of normal load is shown in Fig. 10, for both pairs [0.8, 3.4] and [0.2, 3]. The results show that there is an approximately $15\%$ reduction of $d_{qnet}$, from the beginning to the end of the loading cycle, which confirms the positive correlation between load and channeling. Simulations based on pair [0.2, 3.0] (i.e. CL with the hydraulic aperture set equal to 20% of mechanical aperture) appear to be slightly less channelled than the corresponding simulations based on [0.8, 3.4].

## Discussion

We have assessed a hydro-mechanical experiment using a novel modelling workflow that integrates high-resolution fracture scan data and provides a reliable mechanistic description of the underlying hydro-mechanical processes.

Despite the high accuracy used to characterise the initial fracture aperture roughness, large uncertainty still exists on the initial aperture distribution because of several factors including material loss when the fracture was opened, the difficulty to back reference the two scanned fracture surface datasets and possible rotation occurred during the execution of the flow experiment. Thus, the first step of the modelling workflow has required the definition of an equivalent initial fracture aperture. This was done by bringing close together the two surfaces with a normal displacement. Highly accurate CFD simulations showed that a shift of 0.18 mm is needed to match the experimental flowrate for unloaded conditions. This equivalent fracture aperture, which preserves reasonably well the roughness characteristics of the fracture surfaces, was used as initial condition for the elastic–plastic contact model, which was calibrated to capture the average displacements measured by the four LVDT sensors. The contact model provides a high-resolution description of the evolution of fracture void space at the different loading stages (from unloaded conditions to 8 MPa).

These different aperture distributions, representative of different mechanical loading conditions, were used to discuss the validity of the LCL. This was done by introducing a correction factor (*A*), which implicitly postulates the existence of two different fracture apertures: the mechanical aperture ($\delta _m$), which is the physical separation between the two fracture walls, and the hydraulic aperture ($\delta _h$), which is the fraction of mechanical aperture that is actively transmitting water. A variable exponent for the hydraulic aperture (*B*) was also introduced. The mean squared error (MSE) was computed based on the difference between the computed and the experimental flowrates at the initial (unloaded state) and final stage (load of 8 MPa) of the HM experiment and by varying both *A* and *B* over a certain range. The computed map of MSE shows that optimal results (i.e. low MSE) are obtained for a narrow range of *B*; i.e. from 2.75 to 3.5. If CL is invoked ($B=3.0$), then the hydraulic aperture has to be set equal to 1/5 of the mechanical aperture. This is consistent with several empirical studies that showed that, indeed, only a small part of the mechanical aperture is actively transmitting water^22^. The suggested ratio, $\delta _h/\delta _m=0.2$, is in the upper range estimated from grout injection tests and Posiva Flow Log (PFL) tests performed in grout holes in Olkiluoto ($A \in [0.05, 0.3]$)^12^.

The modified LCL proposed in this study has been validated over a full loading cycle and shows good agreement with the experimental results. The fact that, when applied directly to mechanical aperture, the LCL overestimates flowrates is related to the underlying hypothesis of the approach, which assumes that the second-order partial derivative of velocity perpendicular to the fracture plane is the main component in the Stokes equation^10^. This assumption breaks down when there are abrupt changes in the aperture, as in the fracture being considered. Limited by the few loading steps considered in this work, the proposed empirical parameters (*A*, *B*) are shown to be invariant to changes in load; however, the empirical parameters (*A*, *B*) could be correlated to fracture roughness, e.g., RSD, that is dependent on normal loading. Exploring and quantifying the potential correlation between the empirical parameters and the normal loading remains an important topic for future study.

The correction factor *A* derived in this work is dependent on surface roughness and is, therefore, only applicable to the specific fracture under consideration. However, the related ratio of hydraulic to mechanical aperture falls within the range estimated from in-situ experiments conducted at the Olkiluoto site in Finland. This is one of the few field experiments to date where attempts have been made to establish a relationship between hydraulic and mechanical aperture. It is reasonable to state that in crystalline rocks, the hydraulic aperture of a fracture is only a few tenths of the mechanical aperture. This relationship remains unchanged by variations in normal load unless they significantly alter the internal structure of the void space. Conducting a detailed hydro-mechanical experiment like the one presented in this work may be unfeasible in practical applications. Hence, obtaining fracture and mechanical aperture data from in-situ hydraulic tests is considered the most practical and reliable method.

Another novel aspect of this work is the assessment of the influence of externally applied normal load on channeling. Although it remains difficult to directly observe detailed channeling patterns in the hydro-mechanical experiments using natural rock fracture samples, the numerical simulation results show an evident and significant positive correlation between channeling and normal load. In other words, when a high normal load is applied, the transmissivity of the fracture is reduced, and groundwater flow occurs preferentially in a limited area of the fracture. This feature of stress-dependent channeling has been qualitatively revealed in previous studies, e.g.^21,31,32^. In this work, we further quantitatively demonstrate the impact of normal load on channeling flow in the natural rock fracture using the channeling indicator.

### Supplementary Information

Supplementary Information.

## Data Availability

The data used in this work is owned by the SKB Task Force on Modelling of Groundwater Flow and Transport of Solutes (GWFTS). For further information the reader can obtain contact information from the web site www.skb.se/taskforce.
