# Supplementary material for: Experimental and numerical analysis of flow through a natural rough fracture subject to normal loading
Source: Sci Rep. 2024 Mar 7;14:5587. doi: 10.1038/s41598-024-55751-w (PMC11324755; doi:10.1038/s41598-024-55751-w)
Supplement: Supplementary file 1 — Supplementary Information. [file 41598_2024_55751_MOESM1_ESM.docx]

Supplementary Material for

**Experimental and numerical analysis of flow through a natural rough fracture subject to normal loading**

Paolo Trinchero^1^, Liangchao Zou^2^, Miguel de la Iglesia^1^, Aitor Iraola^1^, Patrick Bruines^3^, Guido Deissmann^4^

^1^ AMPHOS 21 Consulting S.L., c/Vene*ç*uela, 103, 08019 Barcelona, Spain, ^2^ Department of Sustainable Development, Environmental Science and Engineering, KTH Royal Institute of Technology, Stockholm 10044, Sweden. ^3^Swedish Nuclear Fuel and Waste Management Company, Box 3091, SE-169 03 Solna, Sweden, ^3^Gylling GeoSolutions, 3556 Davis Street, Evanston, IL 60203, USA, ^4^Institute of Energy and Climate Research: Nuclear Waste Management (IEK-6) and JARA-CSD, Forschungszentrum Jülich GmbH, 52425 Jülich, Germany.

**Contents of this file**

Figure S1.

Table S1.

**
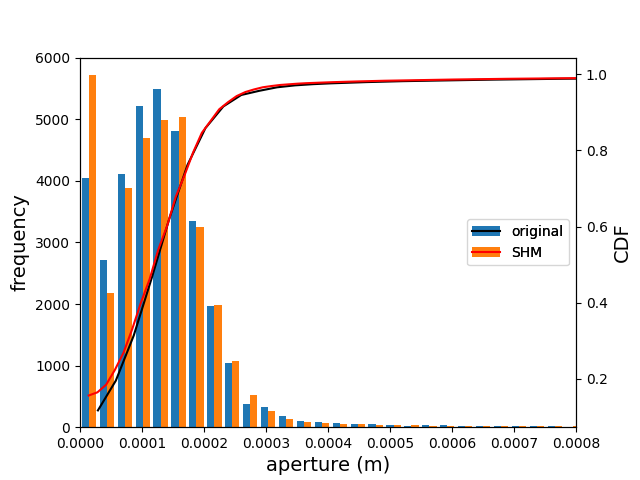
**

**Figure S1**. Histogram and cumulative distribution function (CDF) for the initial fracture aperture as obtained from the fracture scan data of the upper and lower fracture surfaces by applying a shift of 0.18 mm (*original* in the legend) and corresponding aperture distribution as discretised by snappyHexMesh in the CFD simulation (*SHM* in the legend).

| ***p_in_* [Pa]** | ***p_out_* [Pa]** | ***T_w_* [◦C]** | ***T_r_* [◦C]** | ***F* [kN]** | **LVDT_1_ [mm]** | **LVDT_2_ [mm]** | **LVDT_3_ [mm]** | **LVDT_4_ [mm]** | ***Q* [ml/s]** |
| --- | --- | --- | --- | --- | --- | --- | --- | --- | --- |
| 48855.9 | 7007.1 | 19.5 | 19.5 | 0.6 | 3.38·10^-5^ | -1.07·10^-2^ | -1.35·10^-2^ | 2.04·10^-4^ | 1.13·10^-1^ |
| 43840.0 | 6984.6 | 19.5 | 19.5 | 0.6 | 2.08·10^-4^ | -7.35·10^-3^ | -1.06·10^-2^ | 1.81·10^-4^ | 8.81·10^-2^ |
| 38860.6 | 6989.6 | 19.5 | 19.5 | 0.6 | -8.26·10^-5^ | -6.12·10^-3^ | -9.33·10^-3^ | 1.36·10^-4^ | 7.02·10^-2^ |
| 33852.1 | 6995.9 | 19.5 | 19.5 | 0.6 | -2.72·10^-4^ | -5.43·10^-3^ | -8.67·10^-3^ | 1.06·10^-4^ | 5.42·10^-2^ |
| 28873.2 | 6971.6 | 19.4 | 19.5 | 0.6 | -3.82·10^-4^ | -4.45·10^-3^ | -7.62·10^-3^ | 7.94·10^-5^ | 3.96·10^-2^ |
| 48885.9 | 6986.2 | 19.2 | 19.4 | 40.1 | -1.83·10^-2^ | 2.76·10^-2^ | -1.24·10^-3^ | 1.88·10^-3^ | 2.75·10^-2^ |
| 43825.6 | 6986.9 | 19.2 | 19.4 | 40.1 | -1.82·10^-2^ | 2.76·10^-2^ | -1.36·10^-3^ | 1.87·10^-3^ | 2.31·10^-2^ |
| 38795.5 | 7000.3 | 19.2 | 19.4 | 40.1 | -1.81·10^-2^ | 2.76·10^-2^ | -1.34·10^-3^ | 1.88·10^-3^ | 1.85·10^-2^ |
| 33833.5 | 7007.5 | 19.1 | 19.4 | 40.1 | -1.80·10^-2^ | 2.76·10^-2^ | -1.45·10^-3^ | 1.84·10^-3^ | 1.42·10^-2^ |
| 28828.9 | 7005.9 | 19.0 | 19.3 | 40.1 | -1.80·10^-2^ | 2.75·10^-2^ | -1.82·10^-3^ | 1.85·10^-3^ | 1.03·10^-2^ |
| 48818.4 | 6998.4 | 19.0 | 19.3 | 80.1 | -9.66·10^-3^ | 3.37·10^-2^ | 2.92·10^-4^ | 5.28·10^-3^ | 1.81·10^-2^ |
| 43821.9 | 6992.9 | 18.9 | 19.3 | 80.1 | -9.38·10^-3^ | 3.40·10^-2^ | 2.99·10^-4^ | 5.37·10^-3^ | 1.46·10^-2^ |
| 38839.9 | 6988.0 | 18.9 | 19.3 | 80.1 | -9.15·10^-3^ | 3.41·10^-2^ | 3.65·10^-5^ | 5.43·10^-3^ | 1.12·10^-2^ |
| 38821.0 | 7005.7 | 18.9 | 19.3 | 80.1 | -8.83·10^-3^ | 3.46·10^-2^ | 1.81·10^-4^ | 5.51·10^-3^ | 1.11·10^-2^ |
| 48842.2 | 6995.8 | 18.9 | 19.3 | 160.1 | 2.99·10^-4^ | 4.01·10^-2^ | 9.51·10^-3^ | 2.02·10^-2^ | 9.60·10^-3^ |
| 43837.5 | 6994.9 | 18.9 | 19.3 | 160.1 | 7.94·10^-4^ | 4.07·10^-2^ | 9.79·10^-3^ | 2.04·10^-2^ | 8.00·10^-3^ |
| 38853.3 | 6995.4 | 18.9 | 19.3 | 160.1 | 1.15·10^-3^ | 4.12·10^-2^ | 1.01·10^-2^ | 2.05·10^-2^ | 6.30·10^-3^ |
| 48841.2 | 6993.4 | 18.9 | 19.3 | 320.0 | 1.53·10^-2^ | 5.25·10^-2^ | 2.97·10^-2^ | 4.49·10^-2^ | 4.40·10^-3^ |
| 43861.1 | 7000.8 | 19.0 | 19.4 | 320.0 | 1.67·10^-2^ | 5.44·10^-2^ | 3.07·10^-2^ | 4.51·10^-2^ | 3.70·10^-3^ |
| 38813.5 | 6987.9 | 19.2 | 19.6 | 320.0 | 1.86·10^-2^ | 5.69·10^-2^ | 3.21·10^-2^ | 4.54·10^-2^ | 2.90·10^-3^ |
| 38791.0 | 7027.6 | 19.4 | 19.7 | 160.1 | 1.27·10^-2^ | 4.93·10^-2^ | 2.18·10^-2^ | 3.91·10^-2^ | 3.10·10^-3^ |
| 38791.4 | 7031.5 | 19.6 | 19.8 | 80.1 | 1.07·10^-2^ | 4.84·10^-2^ | 1.36·10^-2^ | 2.92·10^-2^ | 3.70·10^-3^ |
| 38793.6 | 7023.0 | 19.7 | 19.8 | 40.1 | 8.13·10^-3^ | 4.57·10^-2^ | 8.39·10^-3^ | 2.28·10^-2^ | 4.20·10^-3^ |
| 38799.2 | 7039.8 | 19.8 | 19.9 | 0.6 | -1.40·10^-2^ | 2.18·10^-2^ | -8.17·10^-3^ | 7.19·10^-3^ | 2.57·10^-2^ |

Table S1. Conditions under which the HM test has been carried out in the direction from face 2 to face 4. *p_in_* and *p_out_* are, respectively, the pressure at the inlet and the outlet boundary; *T_w_* and *T_r_* are, respectively, the temperature in the water measured in vessel V2 and the temperature measured in the rock close to the inflow; *F* is the normal compression force; LVDT1 to LVDT4 are the displacements measured by the corresponding LVDT sensors and *Q* is the measured flowrate.
